# Traditional Chinese mind–body practices and depressive symptoms in university students: a multilevel dose–response meta-analysis

**DOI:** 10.3389/fpsyg.2026.1939249

**Published:** 2026-09-03

**Authors:** Yuming Zhang, Shaoyang Hu, Yonghua Liu, Haoran He, Jiajun Lan, Zhikai Qin

**Affiliations:** 1 Department of Physical Education, Beijing University of Chemical Technology, Beijing, China; 2 Fujian Institute of Education, Fuzhou, China; 3 Xinyang Normal University, Xinyang, China; 4 Capital University of Physical Education and Sports, Beijing, China; 5 School of Physical Education and Sport Science, Fujian Normal University, Fuzhou, China

**Keywords:** Baduanjin, depression, dose–response relationship, meta-analysis, Tai Chi, traditional Chinese mind–body practices, university students

## Abstract

**Objective:**

Numerous meta-analyses have evaluated Tai Chi, Qigong and related traditional Chinese mind–body practices (TCMBPs) for depressive symptoms across broader populations; however, to our knowledge, no previous meta-analysis has focused specifically on university students. We therefore aimed to estimate the average effect of TCMBPs on depressive symptoms in this population using a three-level model that explicitly accounts for dependent effect sizes, and to explore nonlinear dose–response patterns and Bayesian cumulative exposure.

**Methods:**

PubMed, Web of Science, PsycINFO, the Cochrane Library and CNKI were searched from inception to 29 December 2025 for randomized controlled trials. Effect sizes were synthesized with a three-level random-effects model. Heterogeneity, small-study effects and influential estimates were examined using prespecified diagnostics, sensitivity analyses and cluster-robust variance estimation. Subgroup, meta-regression and Bayesian dose–response analyses were treated as exploratory.

**Results:**

Fifteen randomized controlled trials met the eligibility criteria. After influence diagnostics, the pooled estimate indicated lower depressive-symptom scores after traditional Chinese mind–body practice (SMD = −0.56, 95% CI − 0.84 to −0.27; *p* < 0.001), although heterogeneity was substantial and the certainty of evidence was moderate. The estimate was similar with cluster-robust variance estimation. Subgroup patterns differed by practice, comparator and participant characteristics, but most contrasts were supported by few studies. Bayesian models placed the lowest fitted effect near a cumulative dose of 33.7 h; this estimate arose from sparse and uneven exposure data and should not be interpreted as a prescriptive optimum.

**Conclusion:**

Traditional Chinese mind–body practices were associated with a moderate average reduction in depressive symptoms and may be feasible complements to university mental-health programs. Because the evidence was heterogeneous, concentrated in China and of moderate certainty, the findings do not establish equivalent benefits across practices or a recommended dose. Larger, better-reported randomized trials are needed.

**Systematic review registration:**

https://www.crd.york.ac.uk/PROSPERO/view/CRD420251275476, identifier PROSPERO (CRD420251275476).

## Graphical abstract

**Figure:**
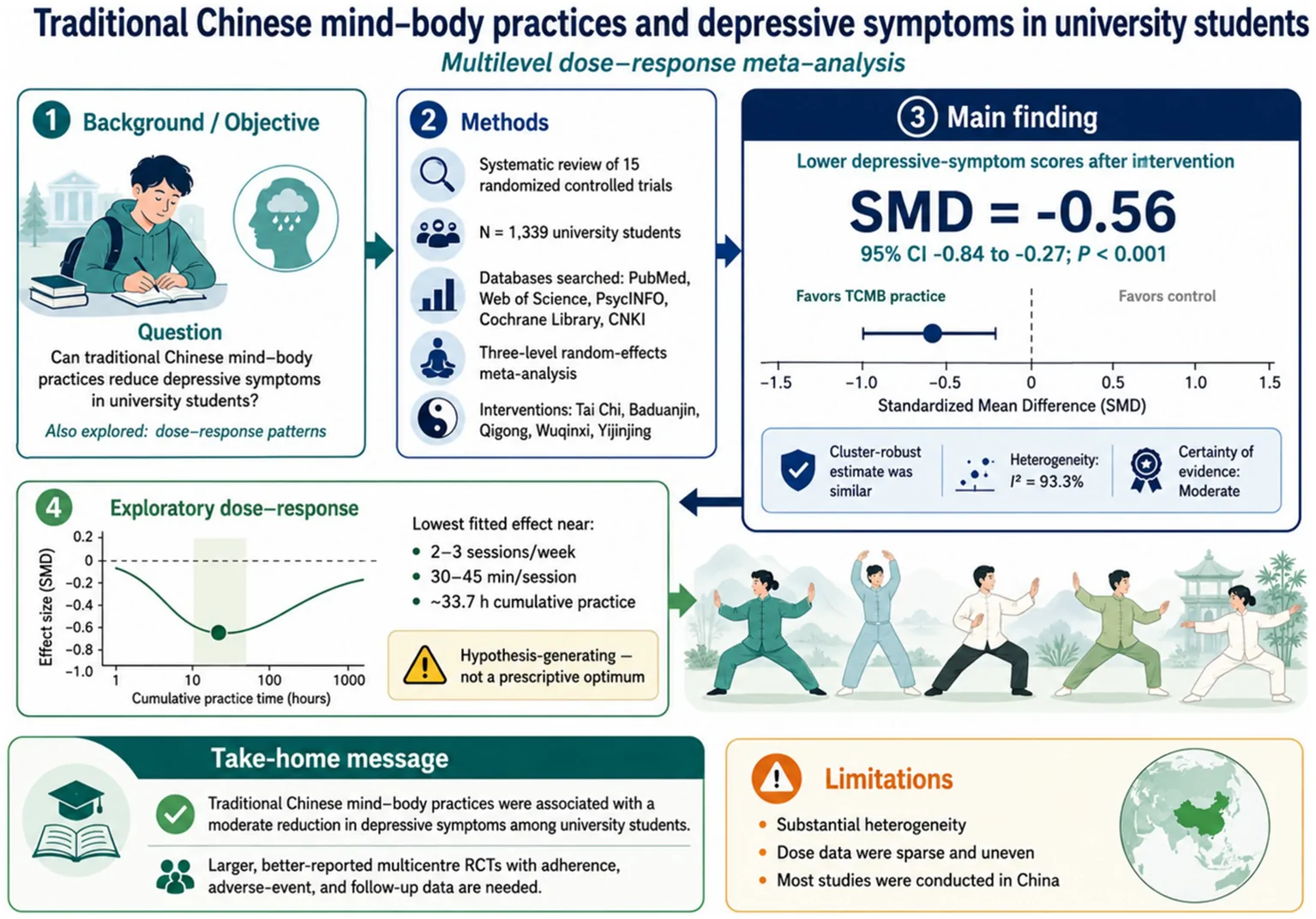

## Introduction

Depressive symptoms are common among university students and often emerge during a period marked by academic, occupational and social transition (Auerbach et al., 2016, 2018). An umbrella review of 8.7 million students estimated prevalences of 35.4% for mild symptoms and 13.4% for severe symptoms (Paiva et al., 2025), while another synthesis reported a pooled prevalence of depression and anxiety symptoms of 33.6% (Li et al., 2022). Young adults aged 18–25 years are at particularly high risk of depression (Lin et al., 2025). Even when symptoms do not meet diagnostic criteria, they can impair daily functioning and identify students who may benefit from accessible preventive support.

Traditional Chinese mind–body practices (TCMBPs)—including Tai Chi, Qigong, Baduanjin, Wuqinxi and Yijinjing—combine coordinated movement, regulated breathing and sustained attention (Jahnke, 2002; Sancier, 1999; Yeung et al., 2018). Contemporary accounts describe these practices as integrating low-to-moderate physical activity with inwardly directed attention (Wang et al., 2023). Proposed mechanisms include modulation of stress-related neuroendocrine pathways and improved emotion regulation (Wu et al., 2023), although these mechanisms remain incompletely established. Their low equipment requirements and adaptability to group teaching make them plausible candidates for campus-based programs.

Previous syntheses suggest that Tai Chi, Qigong and related practices can reduce depressive symptoms, but the magnitude and consistency of benefit remain uncertain. A 2015 meta-analysis reported a moderate effect for Qigong (Cohen’s d approximately −0.48) but no clear effect for Tai Chi (Liu et al., 2015). A review in adolescents reported a larger pooled estimate for Tai Chi and Qigong, accompanied by substantial uncertainty (Liu et al., 2021). Mindfulness-based programs have also improved mental-health outcomes in university students (González-Martín et al., 2023; Ma et al., 2022). Although numerous meta-analyses have evaluated TCMBPs and related interventions across broader populations, to our knowledge, no previous meta-analysis has specifically synthesized their effects on depressive symptoms in university students. A university-specific synthesis is warranted because this population has a distinct developmental and service-delivery context, including academic transition, campus-based physical-education infrastructure and a need for accessible preventive support. Previous reviews also did not establish how program dose relates to depressive-symptom change in university settings.

Existing reviews primarily pooled nominally independent estimates across broader age or clinical groups and did not explicitly model multiple dependent effect sizes nested within the same trial. The novelty of the present review is therefore both population-specific and methodological. We used a three-level meta-analysis to explicitly account for dependent effect sizes and separate sampling, within-study and between-study variance; nonlinear dose–response analysis and Bayesian cumulative-exposure modeling to explore program exposure; and multiple sensitivity analyses, including prespecified influence diagnostics, leave-one-out analysis and cluster-robust variance estimation, to evaluate the robustness of the findings.

## Methods

### Study design

This systematic review followed the PRISMA guidance (Page et al., 2021). The protocol was registered prospectively in PROSPERO (CRD420251275476) before study selection, and the review procedures followed the registered protocol.

### Eligibility criteria

Studies were eligible if they: (1) used a randomized controlled design; (2) evaluated a traditional Chinese mind–body practice in current university students; (3) compared the practice with no intervention, usual care, medication or an active control; (4) assessed depressive symptoms with a standardized instrument; and (5) were available in English or Chinese as full-text reports. We excluded non-experimental and preclinical studies, reviews and meta-analyses, duplicate reports, conference abstracts, unpublished grey literature and unstructured single-session counseling. Reference lists of relevant reviews were screened for additional primary studies.

### Search strategy

We searched PubMed, Web of Science, PsycINFO, the Cochrane Library and CNKI from inception to 29 December 2025. Search terms combined controlled vocabulary and free text for traditional Chinese mind–body practices (including Tai Chi, Baduanjin, Qigong, Zhan Zhuang, Wuqinxi and Yijinjing), university students, depressive symptoms and randomized trials. The PICOS framework defined university students as the population, any eligible mind–body practice as the intervention, inactive or active controls as comparators, change in standardized depression scores as the outcome and randomized controlled trials as the study design. Database-specific strategies are reported in the Supplementary material. The final search update was completed on 29 December 2025; records published after that date were not eligible. Searches and full-text eligibility were restricted to English- and Chinese-language reports, which may have introduced language bias. Complete database-specific strategies are provided in the Supplementary material.

### Study selection

Records were imported into Zotero 7.0 and deduplicated. Two reviewers independently screened titles and abstracts and then assessed potentially eligible full texts against the PICOS criteria. Agreement was quantified with Cohen’s *κ* (Cohen, 1960). Disagreements were resolved by discussion, with a third reviewer available for adjudication. Two reviewers independently extracted participant, intervention, comparator and outcome data using a standardized form and reconciled discrepancies by consensus.

### Data synthesis and statistical methods

Analyses were performed in R version 4.3.3 using the meta, metafor, dosresmeta, diamet, ggplot2 and clubSandwich packages. Outcomes measured on a common scale were summarized as mean differences; outcomes measured with different instruments were summarized as standardized mean differences (SMDs), corrected as Hedges’ g (Nakagawa and Cuthill, 2007).

The primary model was a three-level random-effects meta-analysis fitted with rma.mv and restricted maximum likelihood, separating sampling variance, within-study variance and between-study variance (Assink and Wibbelink, 2016). Small-study effects were examined with funnel plots and Egger’s regression, and trim-and-fill analyses were considered exploratory. Influential estimates were prespecified as those with both |standardized residual| > 2.5 and Cook’s distance > 3 times the mean (Viechtbauer and Cheung, 2010). Robustness was examined with leave-one-out analyses, cluster-robust variance estimation and REML meta-regression (Van Houwelingen et al., 2002). SMDs were calculated with the Hedges–Olkin pooled-standard-deviation formulation (Hedges and Olkin, 2014): $\begin{array}{l} \;\mathrm{SMD}=\frac{M_{\text{Intervention}}-M_{\text{Control}}}{{\mathrm{SD}}_{\text{Pooled}}},\;\;\\ {\mathrm{SD}}_{\text{Pooled}}=\sqrt{\frac{(n_{1}-1){\mathrm{SD}}_{1}^{2}+(n_{2}-1){\mathrm{SD}}_{2}^{2}}{n_{1}+n_{2}-2.}} \end{array}$ .

Bayesian hierarchical dose–response models used restricted cubic splines, weakly informative priors and Markov chain Monte Carlo estimation (Crippa and Orsini, 2016; Hamza et al., 2021). We report posterior medians, 95% credible intervals and posterior predictive intervals, with sensitivity analyses for priors and knot placement. Because exposure data were sparse, all subgroup, meta-regression and dose–response analyses were interpreted as exploratory.

Model specification and reproducibility. Effect sizes were nested within study as random intercepts, sampling variances were treated as known and variance components were estimated by restricted maximum likelihood. Cluster-robust standard errors were calculated at the study level with a small-sample degrees-of-freedom correction. Frequentist nonlinear models were restricted to the observed exposure range. Bayesian spline models used weakly informative priors; convergence was evaluated from trace-plot mixing, R-hat values and effective sample sizes, and sensitivity was assessed by varying priors and knot placement. Influential observations were handled with the prespecified dual criterion and reported sequentially rather than removed on statistical significance alone. Additional specifications, diagnostics and cross-references are provided in the Supplementary material.

### Risk of bias and certainty of evidence

Two reviewers independently assessed each trial with the 2019 Cochrane Risk of Bias 2 tool across randomization, deviations from intended interventions, missing outcome data, outcome measurement and selective reporting (Sterne et al., 2019). Domain-level and overall judgments were classified as low risk, some concerns or high risk; disagreements were resolved by discussion or third-reviewer adjudication. Certainty of evidence for the primary outcome was assessed with GRADE, considering risk of bias, inconsistency, indirectness, imprecision and publication bias (Guyatt et al., 2008). Subgroup and dose–response findings were not used as stand-alone grounds for recommendations.

## Results

### Study selection

**Figure 1:**
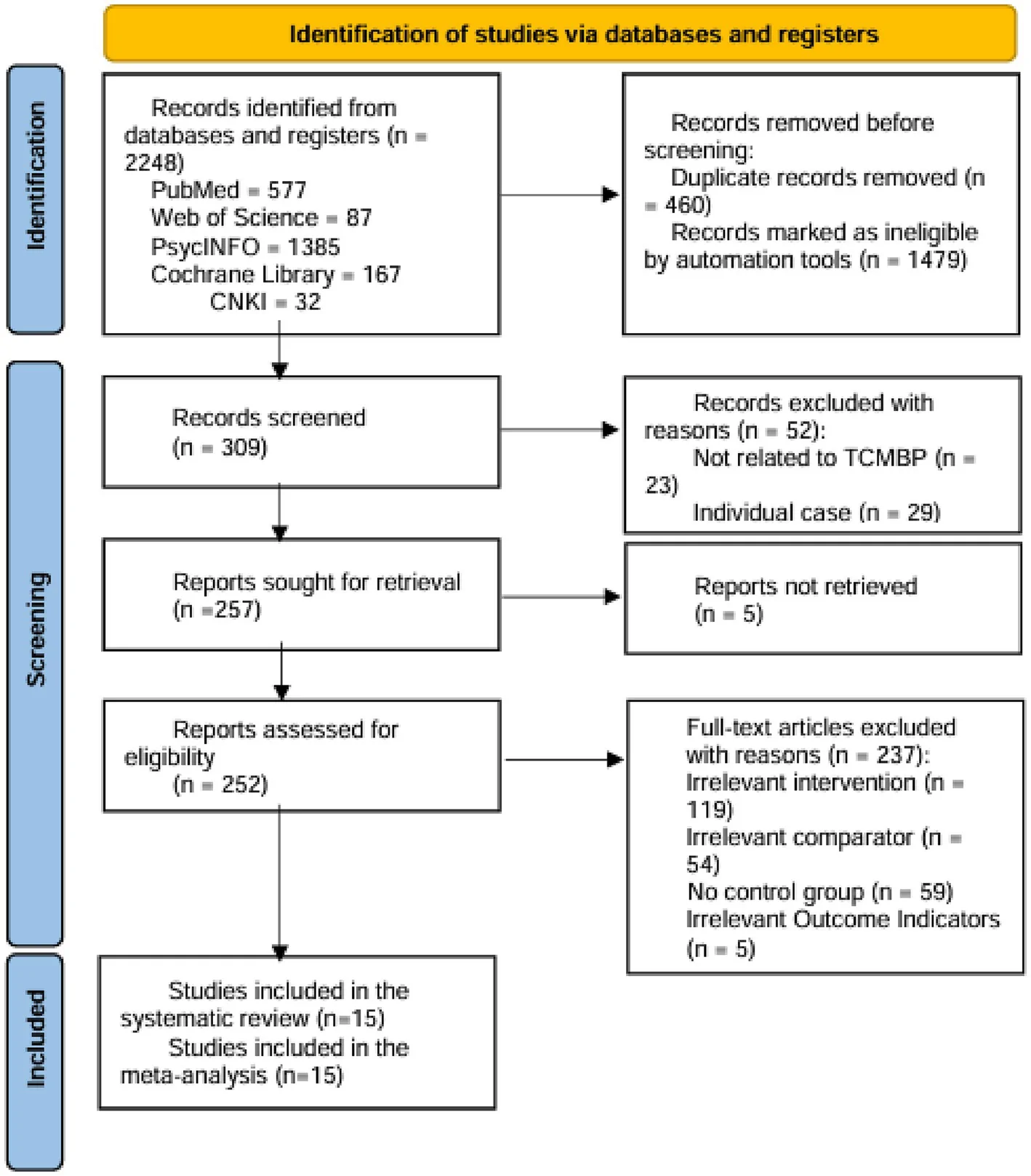
Study-selection flow diagram.

The searches identified 2,248 records. After removal of 460 duplicates and exclusion of 1,479 records during automated and manual prescreening, 309 records underwent title and abstract screening (*κ* = 0.75). Fifty-two were excluded, and 257 reports were sought for retrieval. Five full texts could not be obtained; 252 reports were assessed for eligibility (*κ* = 0.90), of which 237 were excluded for the reasons shown in Figure 1. Fifteen randomized controlled trials were included in the review and contributed quantitative data (Chen et al., 2019; Cheng et al., 2016; Jiao et al., 2021; Ke et al., 2019; Liu and Wang, 2017; Luo, 2021; Qu et al., 2024; Wang, 2021; Wang et al., 2024; Wei et al., 2024; Wei et al., 2017; Zhang et al., 2018, 2023; Zhang, 2021; Zhang and Jiang, 2023).

### Risk of bias of included studies

**Figure 2:**
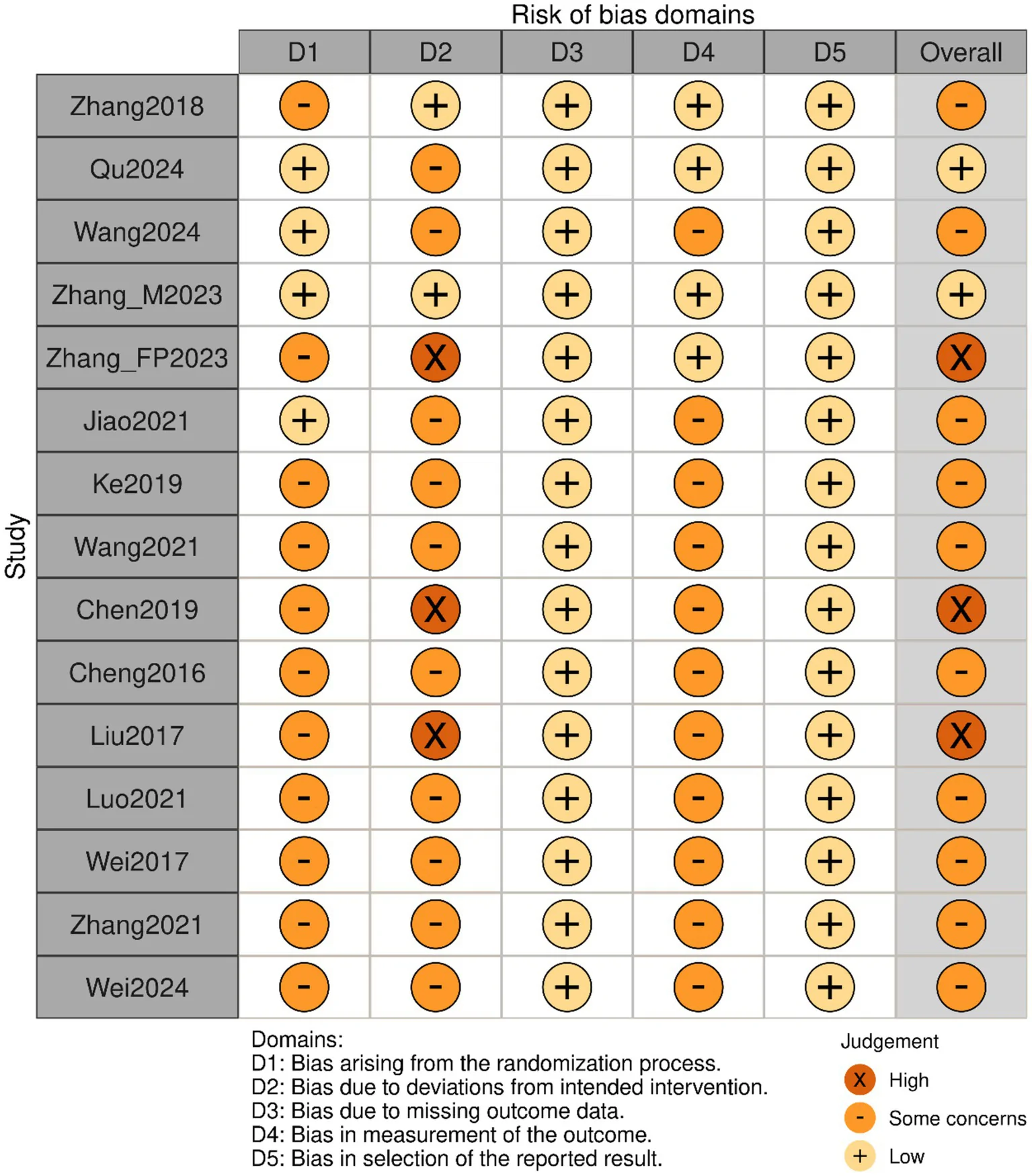
Risk-of-bias judgments by study and domain.

**Figure 3:**
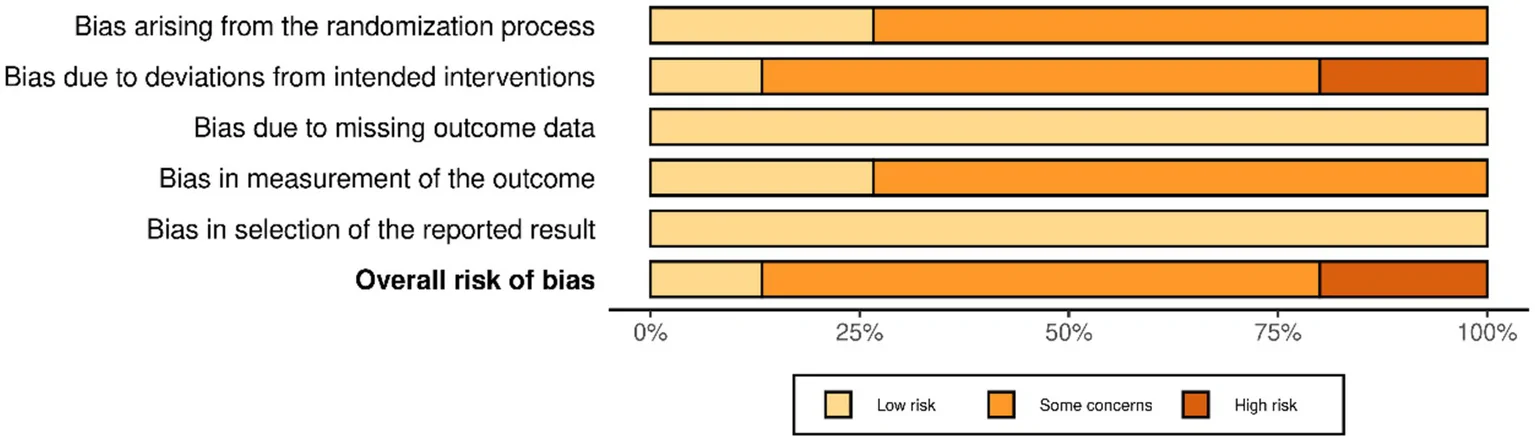
Distribution of risk-of-bias judgments across domains.

Risk-of-bias judgments for the 15 trials are summarized in Figures 2, 3. Agreement between reviewers ranged from moderate to almost perfect across RoB 2 domains: 73.3% for randomization (*κ* = 0.531), 80.0% for deviations from intended interventions (κ = 0.649), 93.3% for missing outcome data (κ = 0.857), 66.7% for outcome measurement (κ = 0.433) and 100% for selective reporting (κ = 1.000). Missing detail on random sequence generation, allocation concealment, blinding and intervention adherence produced the greatest concern, particularly in domains 1 and 2. These limitations informed the sensitivity analyses and the GRADE assessment.

### Characteristics of included studies

The 15 trials enrolled 18–315 participants and were conducted predominantly in university populations aged 18–22 years, although reported ages ranged from approximately 16 to 30 years. Samples included healthy students, students with low physical fitness or sleep problems and students with mild anxiety or depressive symptoms. Interventions comprised Tai Chi, mindfulness-enhanced Tai Chi, Baduanjin, Wuqinxi, Yijinjing and related routines; comparators included no intervention, usual physical-education classes and active exercise programs. Sessions lasted 30–90 min, were delivered one to seven times per week and continued for 1–18 weeks. Depressive symptoms were assessed with PHQ-9, SDS, CES-D, BDI, DASS-21 or SCL-90-R measures. This variation in participants, exposure and outcome measurement was a major source of clinical and methodological heterogeneity (Table 1).

**Table 1:** Characteristics of included randomized controlled trials.

Author/Year	Country	Design	Sample (T/C)	Age range	Subject type	Intervention (T/C)	Protocol (session duration, frequency/week, total duration)	Tools
Zhang et al. (2018)	China	RCT	32/30	16–19	Subthreshold depressed adolescents	MTCC vs. usual PE class	90 min/session, 2×/week, 8 weeks	PHQ-9-Depression subscale
Qu et al. (2024)	China	RCT	69/50	17–22	Healthy university students (beginners)	MTCC vs. Tai Chi	90 min/session, 1×/week, 10 weeks	DASS-21-Depression subscale
Wang et al. (2024)	China	RCT	46/47	18–20	University students with low physical fitness	Baduanjin vs. no-intervention control	60 min/session, 3×/week, 16 weeks	SCL-90-R (depression)
Zhang and Jiang (2023)	China	RCT	39/39	18–20	Female university students	Baduanjin practice vs. no-intervention control	60 min/session, 3×/week, 12 weeks	SCL-90-R (depression)
Zhang et al. (2023)	China	RCT	9/9	18–30	University students with anxiety/depression symptoms	Tai Ji Ba Fa Wu Bu intervention vs. no-intervention control	60 min/session, 5×/week, 8 weeks	SDS
Jiao et al. (2021)	China	RCT	40/40	18–22	Female university students	Traditional Wuqinxi vs. other exercise control	80 min/session, 5×/week, 16 weeks	SCL-90-R (depression)
Ke et al. (2019)	China	RCT	20/17	18–22	Female students with early neck–shoulder syndrome	Baduanjin vs. no-intervention control	45 min/session, 5×/week, 10 weeks	SDS
Wang (2021)	China	RCT	30/30	18–20	Non-PE major female students	Tai Chi vs. aerobic exercise control	60–90 min/session, 3–4×/week, 4 months	SCL-90-R (depression)
Chen et al. (2019)	China	RCT	18/18	18–22	Female students with moderate depression	Tai Chi vs. exercise control vs. usual control	60 min/session, 3×/week, 16 weeks	CES-D
Cheng et al. (2016)	China	RCT	15/14	18–25	Students with mild depression	Fitness Qigong (Wuqinxi) vs. no-intervention control	40–60 min/session, 3×/week, 12 weeks	BDI
Liu and Wang (2017)	China	RCT	40/40	~18–22	Students with a qi-stagnation constitution	Tai Chi vs. blank control	30 min/session, 5×/week, 1 week	SCL-90-R (depression)
Luo (2021)	China	RCT	157/158	18–21	Students with sleep disorders	Tai Chi vs. no-intervention control	30 min/session, 3×/week, 16 weeks	SDS
Wei et al. (2017)	China	RCT	60/60	18–22	University students	Yijinjing vs. no-intervention control	60 min/session, 5×/week, 3 months	SCL-90-R (depression)
Zhang (2021)	China	RCT	30/30	19–22	Students with mild or above depression/anxiety	Baduanjin vs. no-intervention control	90 min/session, 2×/week, 8 weeks	SDS
Wei et al. (2024)	China	RCT	76/76	19–21	First-year students (Guangdong University of Technology)	Baduanjin vs. other PE course control	90 min/session, 1×/week, 18 weeks	PHQ-9

BDI, Beck Depression Inventory; CES-D, Center for Epidemiologic Studies Depression Scale; DASS-21, 21-item Depression Anxiety Stress Scales; MTCC, mindfulness-enhanced Tai Chi Chuan; PHQ-9, Patient Health Questionnaire-9; RCT, randomized controlled trial; SCL-90-R, Symptom Checklist-90-Revised; SDS, Self-Rating Depression Scale; T/C, intervention/control sample size.

### Meta-analysis

**Figure 4:**
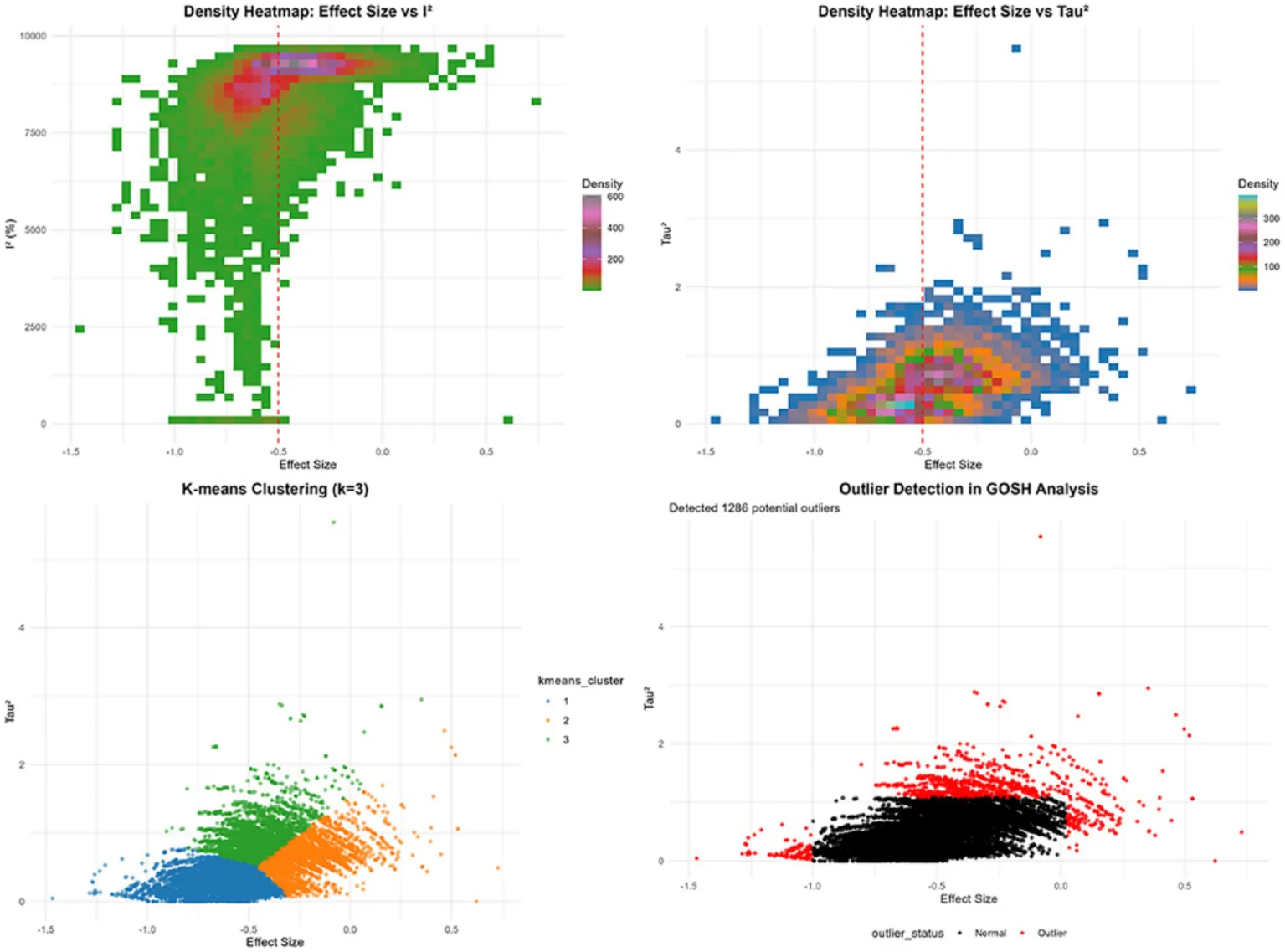
GOSH distributions of pooled effect and heterogeneity across study subsets.

GOSH analysis revealed a broad and multimodal distribution of pooled effects across study subsets (Figure 4). The three-level model before influence-based exclusions yielded an SMD of −0.50 (95% CI − 0.88 to −0.12), with substantial total heterogeneity (I^2^ = 93.3%). Effect estimates covaried with heterogeneity measures across 19,998 subsets, and several clustering algorithms identified a small group of atypical subsets. Because subset-based heterogeneity statistics were unstable at the extremes, these plots were used to locate influential configurations rather than to assign a single causal source of heterogeneity.

**Figure 5:**
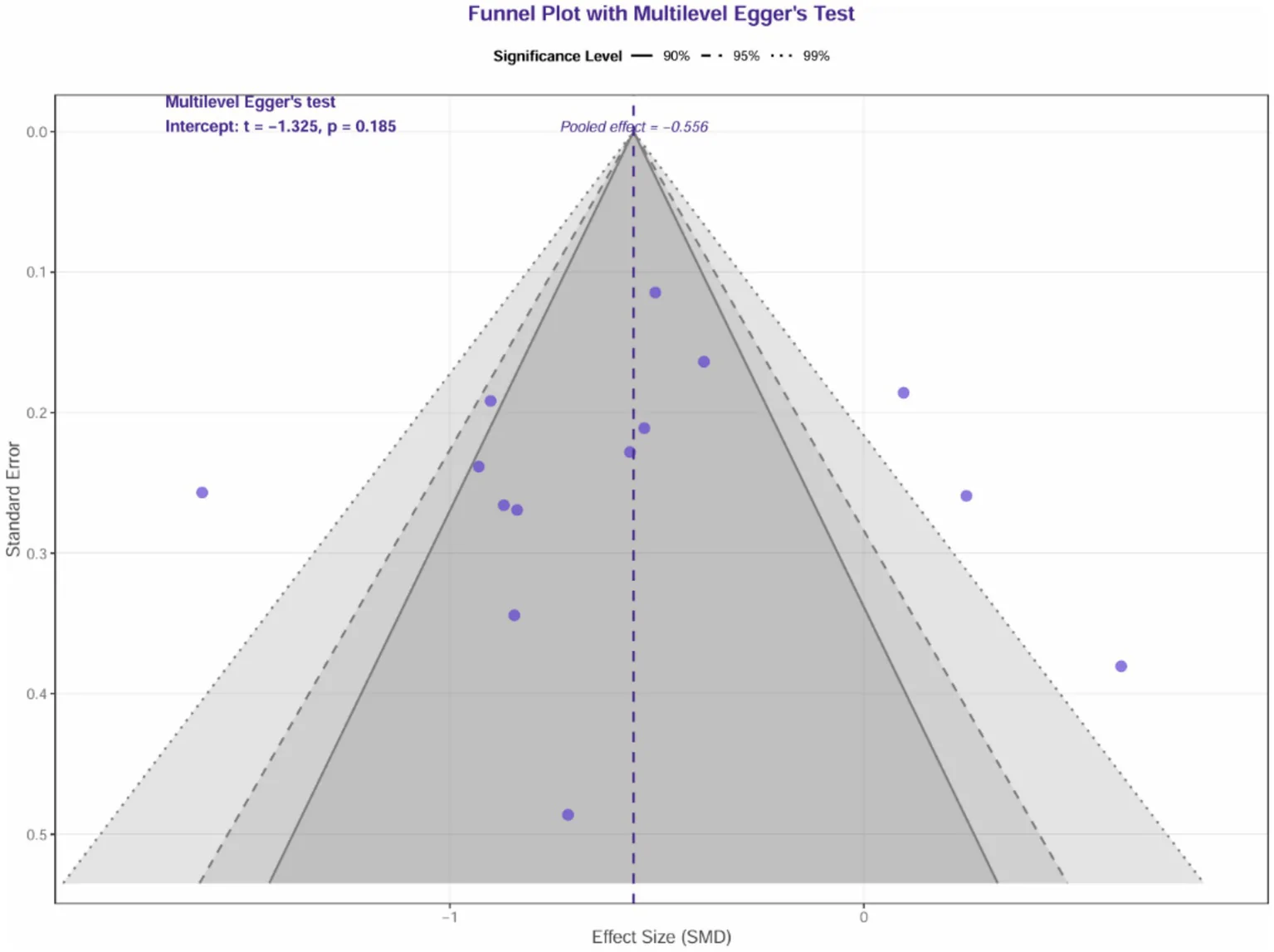
Funnel plot and Egger’s regression for small-study effects.

Egger’s regression did not provide clear evidence of funnel-plot asymmetry (*t* = −1.325, *p* = 0.185; Figure 5) (Gillespie et al., 2012). Influence diagnostics identified one estimate from Chen et al. (2019) that exceeded both the standardized-residual and Cook’s-distance thresholds (Viechtbauer and Cheung, 2010). Removing this estimate shifted the pooled SMD from −0.50 to −0.62. A second estimate from the same trial subsequently met both thresholds, and the trial was therefore excluded from the final pooled model. Liu and Wang, (2017) had a high Cook’s distance but did not exceed the standardized-residual threshold and was retained. Leave-one-out analyses otherwise preserved the direction of the pooled effect.

Chen et al. (2019) was a small three-arm trial of female students with moderate depression that contributed two correlated contrasts against active-exercise and usual-control conditions. After the first estimate met both prespecified influence thresholds and was removed, the second estimate independently met the same thresholds. Retaining only one contrast would therefore leave a trial-level influence and dependence problem that robust standard errors alone do not remove. We excluded the trial from the primary sensitivity-defined pooled model but retained transparent before-exclusion (SMD = −0.50), single-estimate exclusion (SMD = −0.62) and final trial-level exclusion results (SMD = −0.56). The direction of effect was unchanged, indicating that the conclusion did not depend on this exclusion; full sequential diagnostics are cross-referenced in the Supplementary material.

In an exploratory multilevel trim-and-fill analysis of 14 studies, the unadjusted estimate was SMD = −0.56 (95% CI − 0.84 to −0.28). Imputation of two potentially missing studies attenuated the estimate to −0.44 (95% CI − 0.74 to −0.14); refitting the multilevel model produced a similar estimate of −0.44 (95% CI − 0.75 to −0.12). The confidence intervals remained below zero, but the 21.6% relative attenuation indicates that small-study effects cannot be excluded. Given the limited number of studies and substantial heterogeneity, trim-and-fill results were interpreted as sensitivity analyses rather than bias-corrected estimates. The broad random-effects prediction interval crossed the null, consistent with substantial dispersion in effects across settings; accordingly, the pooled SMD is interpreted as an average rather than a prediction of benefit in every context.

**Figure 6:**
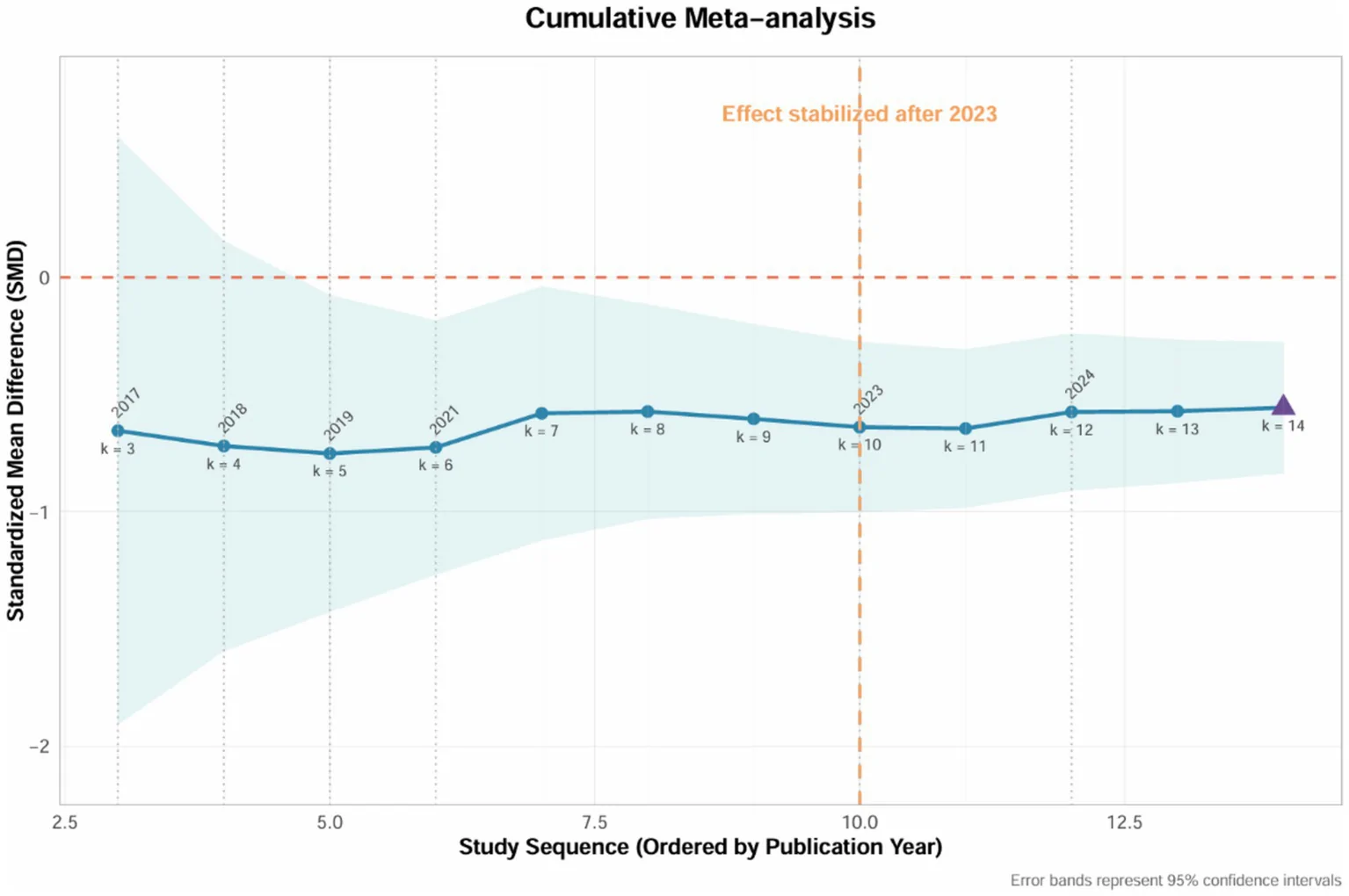
Cumulative meta-analysis ordered by publication year.

**Figure 7:**
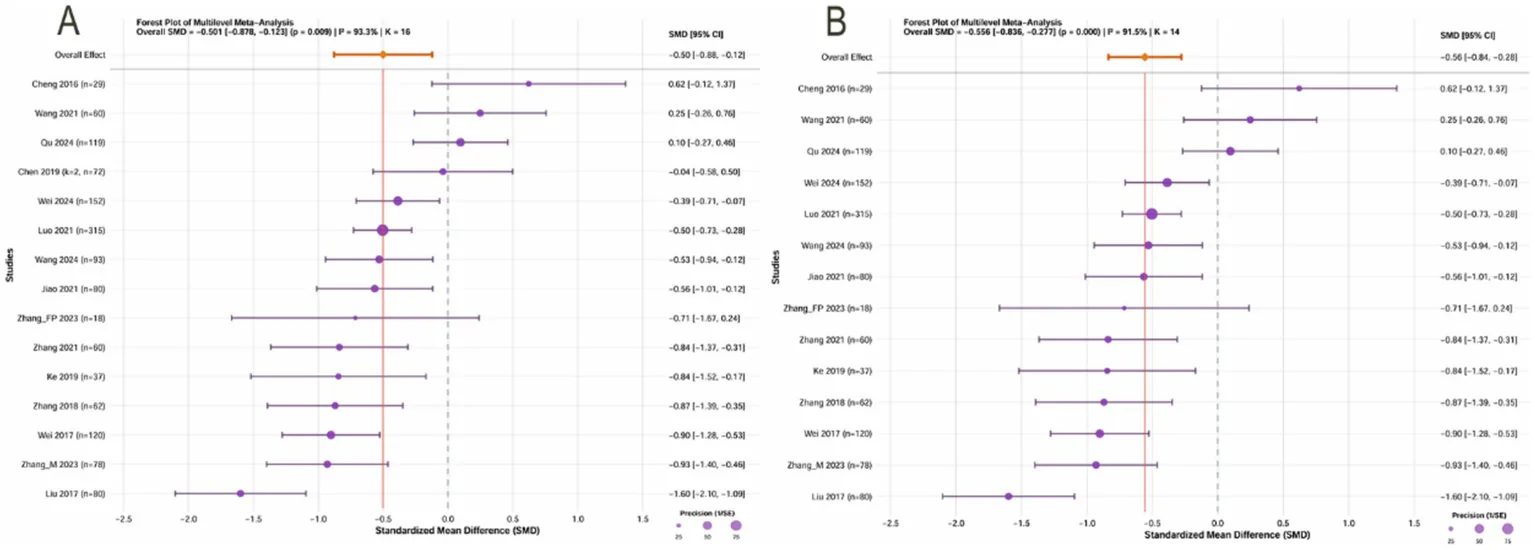
Pooled effects before and after exclusion of influential estimates. **(A)** Forest plot before exclusion of the identified influential/outlying estimates; **(B)** forest plot after exclusion of the identified influential/outlying estimates.

Trial sequential analysis crossed the prespecified monitoring boundary and accumulated information exceeded the modeled required information size. This result supports the stability of the average effect under the assumptions of the sequential model, but it does not resolve between-study heterogeneity, selective reporting or risk of bias. Cumulative meta-analysis showed that the pooled estimate stabilized after the tenth study (Figure 6). Cluster-robust variance estimation also preserved the direction and magnitude of the effect (SMD = −0.56, SE = 0.143, *t*(13.00) = −3.90, *p* = 0.002). The final pooled model therefore indicated lower depressive-symptom scores after traditional Chinese mind–body practice (SMD = −0.56, 95% CI − 0.84 to −0.27; *p* < 0.001; Figure 7). GRADE certainty was moderate, downgraded for limitations in randomization reporting and deviations from intended interventions.

### Subgroup analyses

We prespecified subgroup analyses by intervention form, geographic region, session duration, weekly frequency, RoB 2 classification, program length, comparator, participant subgroup and depression scale. Certainty was summarized with GRADE (Guyatt et al., 2008). Because each comparison contained few studies and multiple moderators were examined, subgroup estimates were treated as exploratory and interpreted primarily through interaction tests rather than within-group significance.

**Figure 8:**
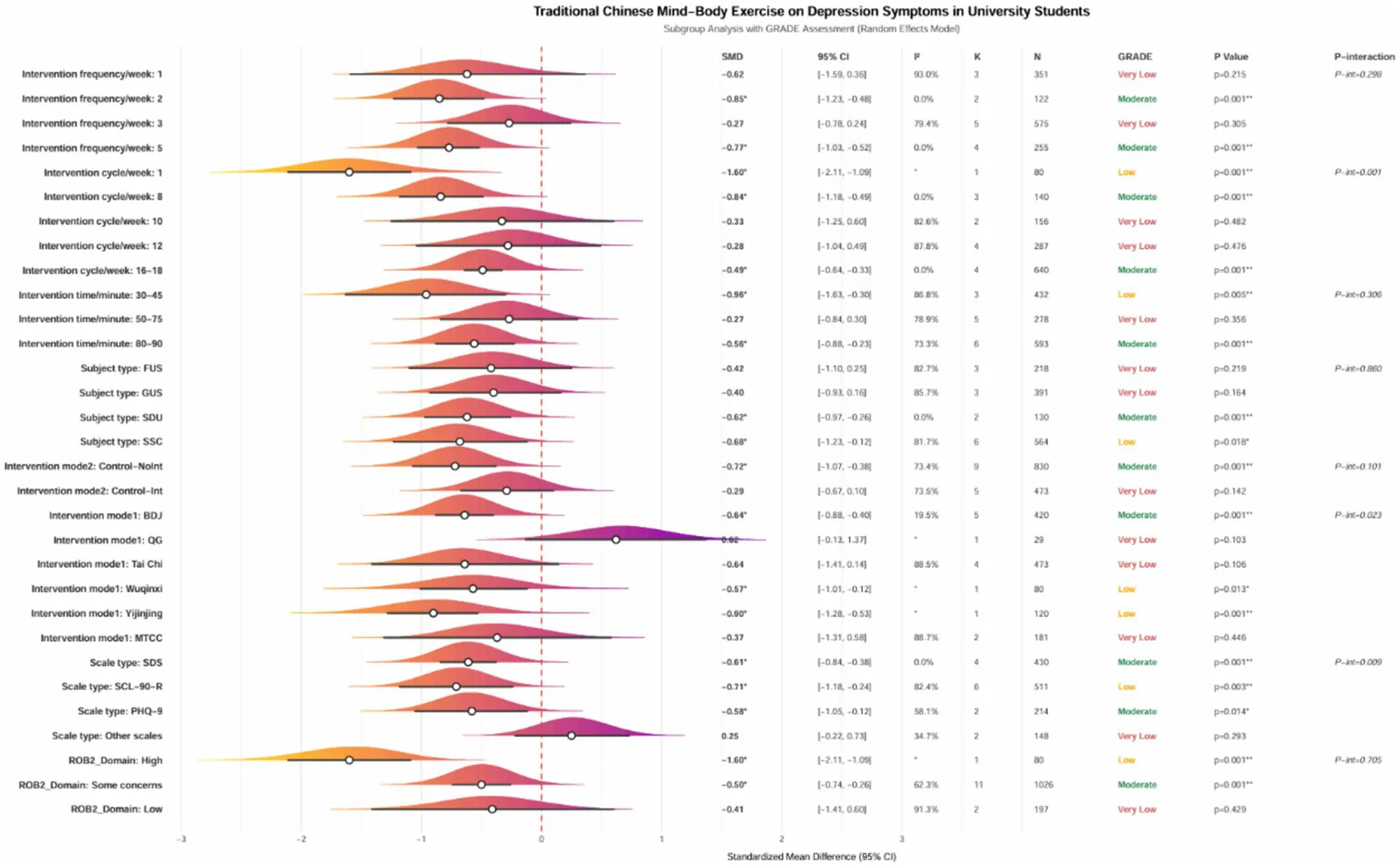
Exploratory subgroup estimates for depressive symptoms. BDJ, Baduanjin; CI, confidence interval; Control-Int, active-intervention control; Control-NoInt, no active intervention; FUS, female university students; GRADE, certainty of evidence; GUS, general university students; MTCC, modified Tai Chi Chuan; OB, obesity; PHQ-9, Patient Health Questionnaire-9; QG, Qigong; RoB 2, Risk of Bias 2; SCL-90-R, Symptom Checklist-90-Revised; SDU, students with low physical fitness; SDS, Self-Rating Depression Scale; SMD, standardized mean difference; SSC, students with specific constitutions. Interaction *p* values test differences between subgroups.

The direction of effect was generally favorable across subgroup analyses (Figure 8), although precision varied. Significant between-subgroup differences were observed for intervention form (Pinteraction = 0.023), program length (Pinteraction = 0.001) and depression scale (Pinteraction = 0.009). Significant effects were found for Baduanjin, Wuqinxi and Yijinjing, but Wuqinxi and Yijinjing each included only one effect size. Significant effects were also observed for 1-, 8- and 16–18-week programs and for SDS, SCL-90-R and PHQ-9. These findings should therefore be interpreted cautiously. Baduanjin and several other named practices had estimates below zero, as did programs delivered two or five times per week and sessions lasting 30–45 or 80–90 min. However, these categories overlapped with participant, comparator and study-quality characteristics, and many estimates were based on only a few trials. The apparent larger effects in students with specific constitutions or low baseline fitness should therefore be considered hypothesis-generating. Comparisons with active interventions did not show a clear additional benefit and were supported by very-low-certainty evidence. These subgroup contrasts should not be read as evidence that any practice is superior because categories contained few studies, interaction tests were underpowered and several moderators were confounded at the study level.

### Linear meta-regression

**Figure 9:**
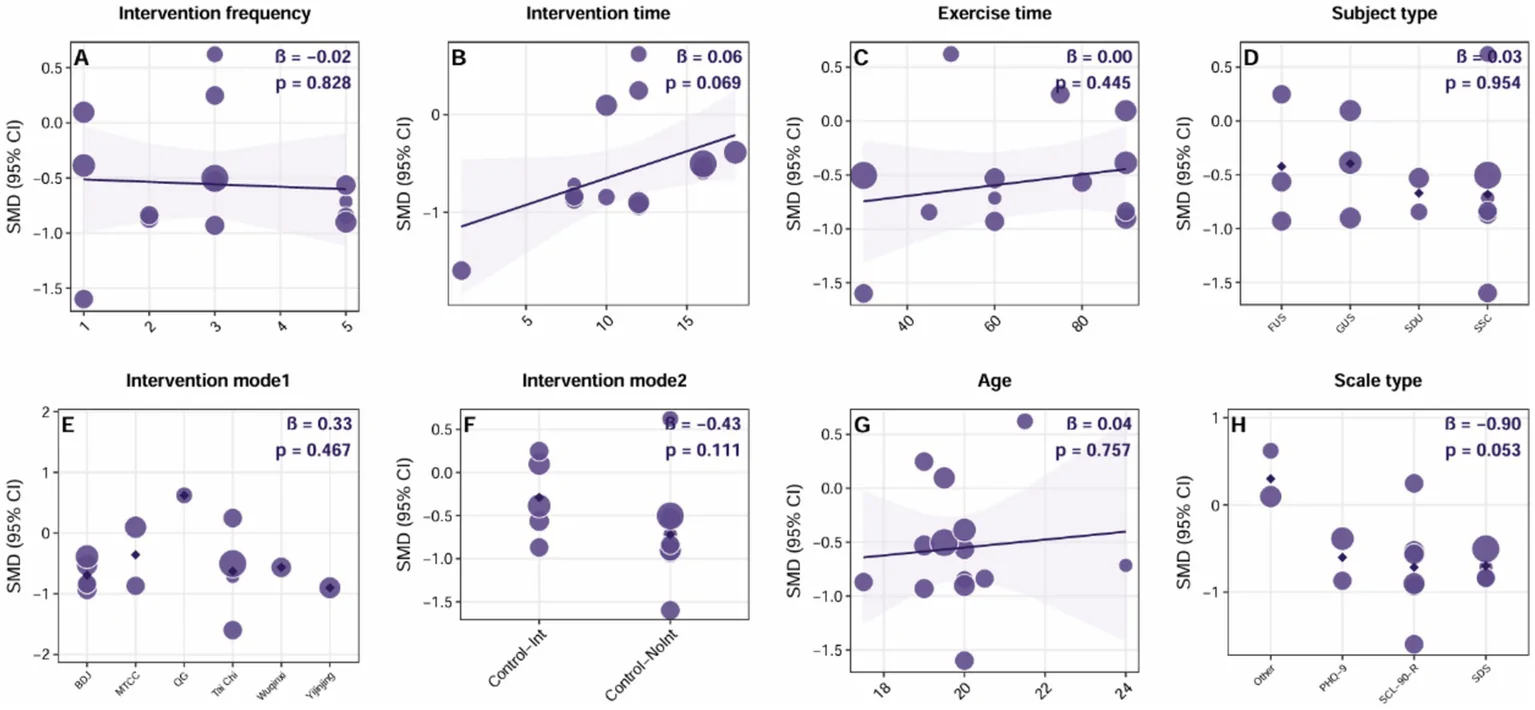
Linear meta-regression of study-level moderators. **(A)** Intervention frequency; **(B)** intervention duration; **(C)** session duration; **(D)** participant type; **(E)** specific traditional Chinese mind-body practice intervention type; **(F)** control condition; **(G)** participant age; and **(H)** depression scale type.

Neither univariable nor multivariable meta-regression identified a clear linear association between effect size and weekly frequency (*β* = −0.02, *p* = 0.828), session duration (*β* = 0.00, *p* = 0.445), participant type (*β* = −0.03, *p* = 0.954), age (*β* = 0.04, *p* = 0.757) or intervention mode (Figure 9). Program length showed a weak positive trend (*β* = 0.06, *p* = 0.069), and depression scale showed a weak negative trend (*β* = −0.90, *p* = 0.053). Neither trend met the prespecified significance threshold, and both may reflect confounding among study-level characteristics.

### Nonlinear regression and dose–response analysis

**Figure 10:**
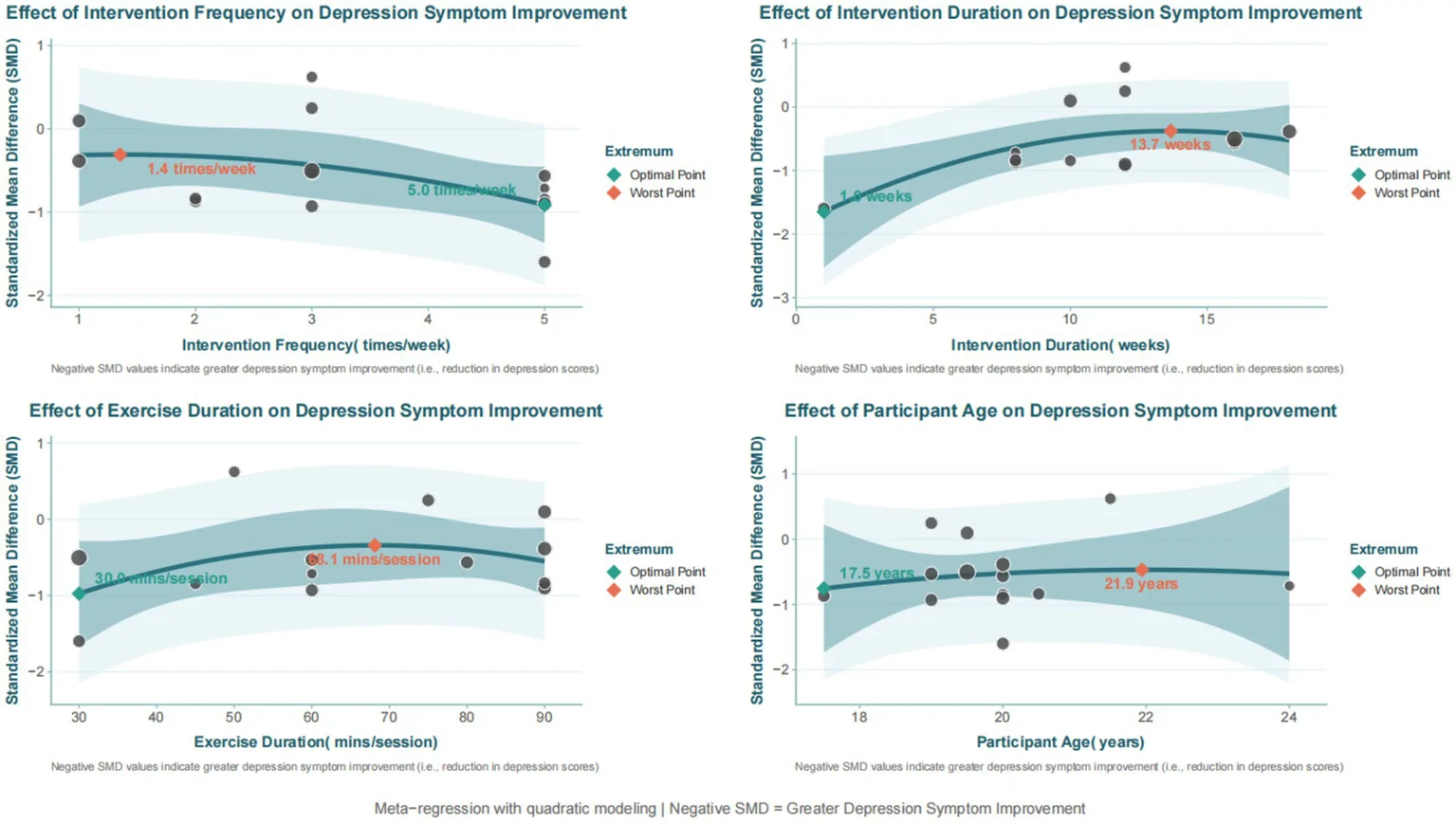
Exploratory nonlinear associations restricted to the observed exposure ranges; fitted minima are not intervention prescriptions.

**Figure 11:**
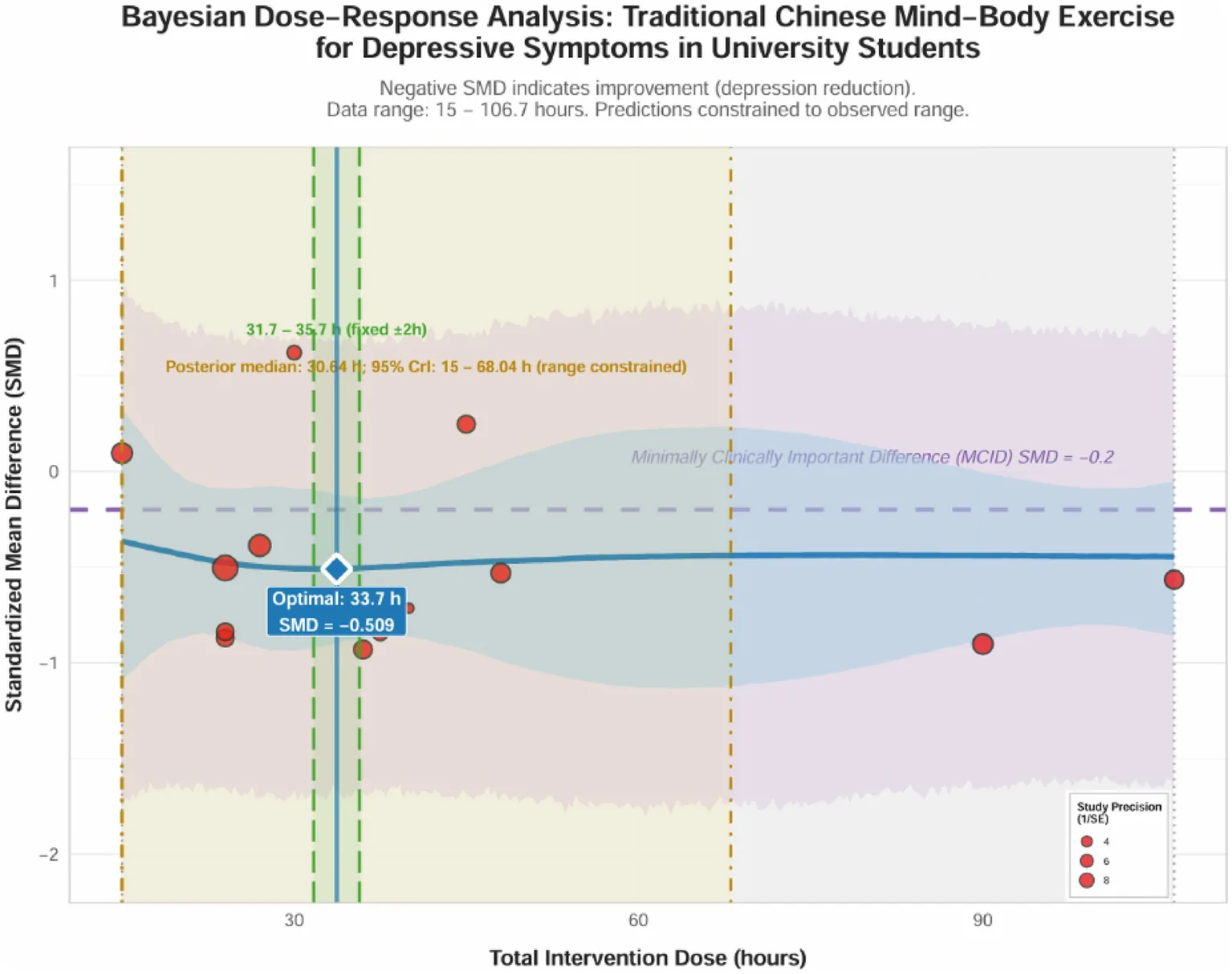
Bayesian cumulative-exposure curve with credible and posterior predictive uncertainty; the fitted minimum is hypothesis-generating.

Nonlinear models suggested that the fitted effect varied across the observed exposure range (Figure 10). The lowest fitted effects occurred near 5 sessions per week and approximately 30 min per session, whereas age contributed little detectable modification. In the Bayesian cumulative-dose model, restricted to interventions longer than 1 week and to the observed range of 15–106.7 h, the posterior median dose was 30.64 h (95% CrI 15.00–68.04 h). The fitted curve reached its minimum near 33.7 h (95% CrI 31.7–35.7 h), at an estimated SMD of approximately −0.51 (Figure 11). Uncertainty widened outside the data-dense region. The 33.7-h value is a model-derived turning point in the fitted curve, not an observed threshold. It is a hypothesis-generating prediction and must not be interpreted as an optimal or recommended dose without prospective dose-ranging randomized trials.

### Integrated evidence synthesis

Traditional Chinese mind–body practices were associated with lower depressive-symptom scores (SMD = −0.56, 95% CI − 0.84 to −0.27), and the direction of effect was preserved across several sensitivity analyses. Confidence in a single pooled value is nevertheless limited by substantial heterogeneity, possible small-study effects and recurring risk-of-bias concerns. Exploratory models placed the lowest fitted effects within moderate exposure ranges, but the available data do not identify a clinically optimal schedule. These estimates are therefore used only to design prospectively powered dose-ranging trials with standardized adherence, comparator and outcome reporting.

## Discussion

Unlike previous meta-analyses conducted across broader or mixed populations, the present review provides, to our knowledge, the first meta-analytic synthesis specifically focused on university students while integrating dependence-aware three-level modeling, nonlinear dose–response analysis, Bayesian cumulative-exposure modeling and multiple sensitivity analyses. The analysis results show that TCMBPs were associated with a moderate reduction in depressive symptoms (SMD = −0.56, 95% CI − 0.84 to −0.27), and sensitivity analyses preserved the direction of the pooled effect. However, RoB 2 identified recurring concerns regarding randomization, allocation concealment, blinding and intervention adherence, particularly in domains 1 and 2. These limitations reduced confidence in the pooled effect and contributed to the overall GRADE rating of moderate certainty. The dose and subgroup analyses generated plausible program characteristics but were supported by fewer studies and greater modeling assumptions. The pooled estimate is broadly consistent with earlier reviews of Qigong and Tai Chi (Liu et al., 2015; Yin and Dishman, 2014) and with a recent synthesis in people aged 15–24 years (Huang et al., 2025). Evidence from other clinical populations also suggests possible mood benefits of Baduanjin (Huang et al., 2024), although differences in population, comparator and outcome scale limit direct comparison.

Variation across trials was not random noise alone: participant characteristics, comparator intensity, intervention form and depression scale were unevenly distributed and often correlated. Students with higher baseline symptom burden or low physical fitness appeared to show larger improvements, a pattern consistent with earlier meta-regression (Yin and Dishman, 2014), but the small subgroup counts preclude firm effect-modifier claims. Similarly, the fitted dose–response curve favored moderate rather than very high exposure, whereas other syntheses have reported benefits with greater weekly volume (Singh et al., 2023; Chekroud et al., 2018). Together, these differences suggest that intervention dose cannot be separated cleanly from who received the program, how it was delivered and what it was compared with.

The apparent larger estimate for Baduanjin should not be interpreted as intrinsic superiority. Baduanjin trials more often used inactive controls and enrolled participants with elevated symptoms or low fitness; comparator intensity and greater baseline room for improvement could therefore magnify standardized effects. Active-control comparisons were closer to the null, suggesting that nonspecific attention and physical activity may explain part of the variation. These are study-level patterns and cannot establish causal moderators. Importantly, neither comparator type (Pinteraction = 0.101) nor participant type (Pinteraction = 0.860) showed significant between-subgroup differences. Therefore, the numerical differences observed across comparator and participant subgroups should be regarded as exploratory rather than evidence of established effect modification. Statistical significance within individual subgroups should not be interpreted as evidence of significant differences between subgroups.

Statistical and clinical heterogeneity should be distinguished. The I^2^ above 90% indicates substantial dispersion beyond sampling error, while variation in interventions, program length and outcome measures suggests genuine clinical and methodological diversity. Subgroup analyses identified significant between-group differences for intervention form (Pinteraction = 0.023), program length (Pinteraction = 0.001) and depression scale (Pinteraction = 0.009), suggesting that these factors may contribute to heterogeneity. Baduanjin provided the more interpretable signal among significant intervention-form subgroups because Wuqinxi and Yijinjing each contained only one effect size, although heterogeneity cannot be attributed to Baduanjin alone.

Several pathways could contribute to the observed symptom changes, including low-intensity physical activity, regulated breathing, attentional training and social participation. Slow coordinated movement and breathing may alter autonomic and stress responses (So et al., 2019), while meditative attention may support interoceptive awareness and emotion regulation (Goyal et al., 2014). Reduced perceived stress may also mediate changes in anxiety and depression (Chan et al., 2020). These mechanisms were not tested in the included trials and should remain hypotheses. For universities, the practical appeal of these practices lies in their low equipment requirements and group-delivery format; implementation should nevertheless include trained instructors, adherence monitoring, adverse-event reporting and referral pathways for students with clinically significant symptoms.

### Practical implications for university mental-health programs

TCMBPs may be considered as optional, low-equipment group activities within a stepped-care framework, not as substitutes for evidence-based assessment or treatment. Pilot programs should use trained instructors, culturally appropriate orientation, active comparison or usual-care monitoring, prospective attendance and home-practice records, standardized symptom measures, adverse-event surveillance and referral pathways for students with clinically significant symptoms. Implementation decisions should prioritize feasibility and acceptability while awaiting confirmatory effectiveness and dose-ranging trials.

### External validity and cultural context

Because almost all trials were conducted in China, the observed acceptability, attendance and symptom response may depend partly on cultural familiarity with Tai Chi and related practices. Effects in Western universities cannot be assumed to match the pooled estimate.

In settings where TCMBPs are less familiar, expectancy, instructor credibility, language, program framing and access to qualified teachers may alter engagement and outcomes. Multinational randomized trials should therefore test culturally adapted delivery against credible active controls and report recruitment, adherence, acceptability and adverse events before broad implementation is recommended.

### Limitations

Several limitations define the boundary of these findings. Total heterogeneity exceeded 90%, reflecting variation in participants, interventions, comparators and outcome instruments. Many trials incompletely reported sequence generation, allocation concealment, blinding and adherence. Dose variables were reconstructed from program schedules and rarely captured attendance or unsupervised practice, weakening causal interpretation of the dose–response curve. Long-term follow-up and adverse-event reporting were uncommon. Finally, most studies were conducted in China; transferability to other university systems and cultural settings remains uncertain. The standardized mean difference also does not map directly to a single patient-level minimal clinically important difference across the six depression instruments. Adherence and adverse events were too inconsistently reported to determine whether the intended exposure was received or whether harms were fully captured.

## Conclusion

Traditional Chinese mind–body practices were associated with a moderate average reduction in depressive symptoms among university students, but the standardized effect should not be interpreted as a uniform or directly patient-level clinically important change across all practices and scales. The nonlinear and Bayesian analyses define testable exposure ranges for future trials, not a recommended prescription. Multicentre and multinational randomized studies should compare standardized programs with credible active controls, prospectively measure adherence and adverse events, and assess persistence of benefit before broad implementation.

## Data Availability

The original contributions presented in the study are included in the article/Supplementary material, further inquiries can be directed to the corresponding author.
