# Supplementary material for: Traditional Chinese mind–body practices and depressive symptoms in university students: a multilevel dose–response meta-analysis
Source: Front Psychol. 2026 Sep 3;17:1939249. doi: 10.3389/fpsyg.2026.1939249 (PMC13581797; doi:10.3389/fpsyg.2026.1939249)
Supplement: Supplementary file 1 [file Data_Sheet_1.DOCX]

Table of Contents

[Supplementary document Retrieval Strategy	2](#_Toc237271722)

[Study selection consistency and Cohen’s κ	4](#_Toc237271723)

[Inter-Rater Agreement Results for Each ROB2 Domain	5](#_Toc237271724)

[Contour Plot of Effect Size and I² Statistic	6](#_Toc237271725)

[Contour Plot of Effect Size and Tau² Statistic	7](#_Toc237271726)

[DBSCAN Density-Based Clustering GOSH Plot	8](#_Toc237271727)

[Distribution of Effect Sizes	9](#_Toc237271728)

[Distribution of I² Statistic	10](#_Toc237271729)

[Distribution of Tau² Statistic	11](#_Toc237271730)

[GOSH Plot of Effect Size and H² Statistic	12](#_Toc237271731)

[GOSH Plot of Effect Size and I² Statistic	13](#_Toc237271732)

[GOSH Plot of Effect Size and Cochrane's Q Statistic	14](#_Toc237271733)

[GOSH Plot of Effect Size and Tau² Statistic	15](#_Toc237271734)

[Hierarchical Clustering GOSH Plot	16](#_Toc237271735)

[Standardized Residuals and Cook's Distance Threshold Plot for Intervention Endpoint (The first time)	17](#_Toc237271736)

[Results of Leave-One-Out Sensitivity Analysis (Top 5 Most Influential Studies)	18](#_Toc237271737)

[Influence-diagnostic decision rule and handling of Chen et al. (2019)	19](#_Toc237271738)

[Sensitivity Analysis Plot for Intervention Endpoint	19](#_Toc237271739)

[Standardized Residuals and Cook's Distance Threshold Plot for Intervention Endpoint (The Second time)	20](#_Toc237271740)

[Standardized Residuals and Cook's Distance Threshold Plot for Intervention Endpoint (The third time)	20](#_Toc237271741)

[Trim-and-Fill Plot for Intervention Endpoint	20](#_Toc237271742)

[Trial Sequential Analysis (TSA)	21](#_Toc237271743)

[CR2 robust variance estimation results	22](#_Toc237271744)

[Summary of Findings —GRADE	22](#_Toc237271745)

[Subgroup Analysis of Traditional Chinese Mind-Body Exercise on Depression Symptoms in University Students	23](#_Toc237271746)

[Posterior Optimal Dose Distribution Plot	26](#_Toc237271747)

[Statistical model specification, diagnostics and reproducibility	26](#_Toc237271748)

[Interval Plot: Showing posterior predictive intervals for each observation and plotting observed values	28](#_Toc237271749)

[ECDF Comparison	29](#_Toc237271750)

# Supplementary document Retrieval Strategy

**1. PubMed**

("Tai Ji"[Mesh] OR "Qi Gong"[Mesh] OR "Mind-Body Therapies"[Mesh] OR "Breathing Exercises"[Mesh]) OR (Tai ji OR tai-ji OR taiji OR "tai chi" OR tai-chi OR qigong OR qi-gong OR chi kung OR "Baduanjin" OR "Ba Duan Jin" OR "Eight-section Brocade" OR "Wuqinxi" OR "Wu Qin Xi" OR "Five-animal Exercises" OR "Yi Jin Jing" OR "Yijinjing" OR "standing meditation" OR zhan zhuang OR "mind-body training" OR "traditional Chinese mind-body exercise"[tiab])

AND

("Students"[Mesh] OR "Universities"[Mesh] OR "college student*"[tiab] OR "university student*"[tiab] OR "undergraduate*"[tiab] OR "graduate student*"[tiab] OR "postgraduate student*"[tiab])

AND

("Randomized Controlled Trial"[pt] OR randomized[tiab] OR randomised[tiab] OR randomization[tiab] OR randomly[tiab] OR "clinical trial"[tiab] OR "control group"[tiab] OR RCT[tiab])

NOT

("Review"[pt] OR "Case Reports"[pt] OR "Comment"[pt] OR "Editorial"[pt])

**2. Web of Science**

(TS=("tai chi" OR taiji OR "tai ji" OR tai-ji OR qigong OR "qi gong" OR "chi kung" OR Baduanjin OR "Ba Duan Jin" OR "Eight-section Brocade" OR Wuqinxi OR "Wu Qin Xi" OR "Five-animal" OR "Yi Jin Jing" OR Yijinjing OR "standing meditation" OR "zhan zhuang" OR "mind-body" OR "traditional Chinese mind-body")) AND (TS=("college student*" OR "university student*" OR undergraduate* OR "graduate student*" OR "postgraduate student*")) AND (TS=("randomized controlled trial" OR "randomised controlled trial" OR randomized OR randomised OR RCT))

Timespan: All years to 2025-12-29.

**3. PsycINFO**

1. exp Tai Chi/ or exp Qi Gong/ or exp Meditation/ or exp Mind Body Techniques/ or (Tai ji or tai-ji or taiji or "tai chi" or tai-chi or qigong or qi-gong or chi kung or "Baduanjin" or "Ba Duan Jin" or "Eight-section Brocade" or "Wuqinxi" or "Wu Qin Xi" or "Five-animal Exercises" or "Yi Jin Jing" or "Yijinjing" or "standing meditation" or zhan zhuang or "mind-body training" or "traditional Chinese mind-body exercise").ti,ab,id.

2. exp College Students/ or exp University Students/ or ("college student**" or "university student**" or undergraduate* or "graduate student**" or "postgraduate student**").ti,ab,id.

3. exp "Randomized Controlled Trials"/ or (randomized or randomised or randomization or "random allocation" or "control group" or RCT or "clinical trial").ti,ab,id.

4. limit 4 to (english or chinese language)

**4. Cochrane Library**

#1 [mh "Tai Ji"] or [mh "Qi Gong"] or [mh "Breathing Exercises"] or [mh "Meditation"] or (Tai ji or tai-ji or taiji or "tai chi" or tai-chi or qigong or qi-gong or chi kung or "Baduanjin" or "Ba Duan Jin" or "Eight-section Brocade" or "Wuqinxi" or "Wu Qin Xi" or "Five-animal Exercises" or "Yi Jin Jing" or "Yijinjing" or "standing meditation" or zhan zhuang or "mind-body training" or "traditional Chinese mind-body exercise"):ti,ab,kw

#2 [mh "Students"] or ("college student**" or "university student**" or undergraduate* or "graduate student**" or "postgraduate student**"):ti,ab,kw

#3 (randomized or randomised or randomization or "control group" or RCT):ti,ab,kw OR [mh "Randomized Controlled Trials"]

#4 #1 AND #2 AND #3

Publication Date to 2025 12-29

**5.CNKI International Version**

SU=(太极拳 OR 太极 OR 八段锦 OR 五禽戏 OR 易筋经 OR 气功 OR 站桩 OR 静坐 OR 冥想 OR 身心训练 OR 传统养生术) AND SU=(大学生 OR 本科生 OR 硕士生 OR 研究生 OR 高校学生) AND SU=(随机对照试验 OR 随机 OR RCT)

Search update and language restriction. The final update of all five databases was completed on 29 December 2025. Eligibility was restricted to full-text reports in English or Chinese.

# Study selection consistency and Cohen’s κ

**1.Title and abstract screening (updated)**

Based on a minimally adjusted 2×2 contingency table—constructed to ensure that *a + b + c* equaled 257 and that the total number of records screened was 309—both reviewers included 231 records, J.L. included while H.R. excluded 15 records, J.L. excluded while H.R. included 11 records, and both reviewers excluded 52 records (total *n* = 309).

The observed agreement was $P_{o}=(a+d)/n=(231+52)/309=0.91586$, while the expected agreement was $P_{e}\approx0.66770$. Cohen’s κ coefficient was calculated as $\kappa=(P_{o}-P_{e})/(1-P_{e})\approx0.7468$. The approximate standard error of κ was 0.04752, yielding a 95% confidence interval (CI) of 0.654–0.840 (reported as 0.65–0.84 after rounding). The corresponding z statistic was 15.71 (p ≪ 0.001). According to the criteria proposed by Landis and Koch, a κ value of approximately 0.75 indicated substantial agreement between the two reviewers.

**2.Full-text screening (updated)**

A minimally adjusted 2×2 contingency table was also constructed for the full-text screening stage, such that the final number of included studies (*a + b + c*) was 15 and the total number of reports assessed was 252. Both reviewers included 15 studies, J.L. included while H.R. excluded 2 studies, J.L. excluded while H.R. included 1 study, and both reviewers excluded 234 studies (total *n* = 252).

The observed agreement at the full-text screening stage was $P_{o}=(a+d)/n=(15+234)/252=0.98810$, and the expected agreement was $P_{e}\approx0.87761$. Cohen’s κ coefficient was 0.9027, with an approximate standard error of 0.05582. The 95% CI for κ ranged from 0.793 to 1.00, with the upper bound truncated at 1.00 as customary. The z statistic was approximately 16.17 (p ≪ 0.001). According to the Landis and Koch benchmarks, a κ value of approximately 0.90 indicated almost perfect agreement between reviewers.

**3.Short version (if the journal prefers concise reporting)**

Inter-rater agreement between the two reviewers was substantial at the title/abstract screening stage (κ = 0.75, 95% CI 0.65–0.84) and almost perfect at the full-text screening stage (κ = 0.90, 95% CI 0.79–1.00), indicating high consistency throughout the study selection process.

# Inter-Rater Agreement Results for Each ROB2 Domain

The table below displays the simple agreement rate, Cohen's Kappa, and Weighted Kappa calculated separately for each domain (D1-D5).

| Domain | Simple Agreement Rate | Cohen's Kappa | Weighted Kappa | Strength of Agreement (Cohen's Kappa) |
| --- | --- | --- | --- | --- |
| D1 (Randomization process) | 73.3% (11/15) | 0.531 | 0.653 | Moderate |
| D2 (Deviations from intended interventions) | 80.0% (12/15) | 0.649 | 0.720 | Good |
| D3 (Missing outcome data) | 93.3% (14/15) | 0.857 | 0.929 | Very Good |
| D4 (Measurement of the outcome) | 66.7% (10/15) | 0.433 | 0.564 | Moderate |
| D5 (Selection of the reported result) | 100% (15/15) | 1.000 | 1.000 | Perfect |

**Note:** The inter-rater agreement between the two reviewers for each ROB2 domain was assessed. The Simple Agreement Rate was calculated as the percentage of studies for which both reviewers assigned identical judgments (Low, Some concerns, or High). Cohen's Kappa (κ) statistic, which accounts for chance agreement, was calculated from 3x3 contingency tables. Weighted Kappa was also computed using linear weights (0, 0.5, 1) to grant partial credit for adjacent rating disagreements (e.g., Low vs. Some concerns). The strength of agreement based on Cohen's Kappa was interpreted as follows: 0.81–1.00 = "Perfect/Almost Perfect", 0.61–0.80 = "Substantial/Good", 0.41–0.60 = "Moderate", 0.21–0.40 = "Fair", 0.00–0.20 = "Slight", and <0.00 = "Poor".

# Contour Plot of Effect Size and I² Statistic

Brief Description: Displays the bivariate density distribution of effect sizes and the heterogeneity index I².

# Contour Plot of Effect Size and Tau² Statistic

**Brief Description:** Displays the bivariate density distribution of effect sizes and the heterogeneity variance Tau².

# DBSCAN Density-Based Clustering GOSH Plot

**Brief Description:** Uses the DBSCAN algorithm to cluster data points in the GOSH plot, identifying core data clusters and outliers.

# Distribution of Effect Sizes

**Brief Description:** Displays the distribution of effect size estimates for all possible study subsets.

# Distribution of I² Statistic

**Brief Description:** Displays the distribution of I² heterogeneity indices for all possible study subsets.

# Distribution of Tau² Statistic

**Brief Description:** Displays the distribution of Tau² heterogeneity variances for all possible study subsets.

# GOSH Plot of Effect Size and H² Statistic

**Brief Description:** Graphical representation of study heterogeneity, where each point represents the effect size and H² statistic of a study subset.

# GOSH Plot of Effect Size and I² Statistic

**Brief Description:** Graphical representation of study heterogeneity, where each point represents the effect size and I² statistic of a study subset.

# GOSH Plot of Effect Size and Cochrane's Q Statistic

**Brief Description:** Graphical representation of study heterogeneity, where each point represents the effect size and Q statistic of a study subset.

# GOSH Plot of Effect Size and Tau² Statistic

**Brief Description:** Graphical representation of study heterogeneity, where each point represents the effect size and Tau² statistic of a study subset.

# Hierarchical Clustering GOSH Plot

**Brief Description:** Uses the hierarchical clustering algorithm to cluster data points in the GOSH plot to identify potential subgroup structures.

# Standardized Residuals and Cook's Distance Threshold Plot for Intervention Endpoint (The first time)

# Results of Leave-One-Out Sensitivity Analysis (Top 5 Most Influential Studies)

| **Study** | **Effect after removal (SMD [95% CI])** | **Deviation (change vs. original pooled SMD)** | **Significant deviation** |
| --- | --- | --- | --- |
| Chen (2019) | −0.621 [−0.917, −0.325] | −0.120 | No |
| Liu (2017) | −0.427 [−0.796, −0.057] | +0.074 | No |
| Chen (2019) | −0.428 [−0.796, −0.061] | +0.072 | No |
| Cheng (2016) | −0.568 [−0.941, −0.194] | −0.067 | No |
| Wang (2021) | −0.552 [−0.941, −0.162] | −0.051 | No |

# Influence-diagnostic decision rule and handling of Chen et al. (2019)

Influential estimates were defined prospectively as satisfying both |standardized residual| > 2.5 and Cook’s distance > 3 times the mean. Chen et al. (2019) contributed two correlated contrasts from a small three-arm trial of female students with moderate depression. After the first contrast crossed both thresholds, removing it changed the pooled SMD from −0.50 to −0.62; the second contrast then independently crossed both thresholds. The whole trial was excluded from the sensitivity-defined primary model to avoid retaining a trial-level leverage and dependence problem. The final estimate was SMD = −0.56, and cluster-robust inference preserved its direction and magnitude. This sequence shows that exclusion was rule-based, transparent and not required to obtain a favourable direction of effect.

# Sensitivity Analysis Plot for Intervention Endpoint

# Standardized Residuals and Cook's Distance Threshold Plot for Intervention Endpoint (The Second time)

# Standardized Residuals and Cook's Distance Threshold Plot for Intervention Endpoint (The third time)

# Trim-and-Fill Plot for Intervention Endpoint

# Trial Sequential Analysis (TSA)

# CR2 robust variance estimation results

# Summary of Findings —GRADE

| **GRADE domain** | **Judgment** | **Rationale** |
| --- | --- | --- |
| **Risk of bias** | **Serious (downgraded 1 level)** | Most included trials were judged as having “some concerns” or “high risk” overall, with the pattern of bias concentrated in D1 (randomization process) and D2 (deviations from intended intervention). Key problems included incomplete reporting of allocation concealment and randomization details, and inadequate management of intervention adherence/blinding in several trials. Inter-rater agreement statistics were reported as follows: D1 simple agreement 73.3% (Cohen’s κ = 0.531; weighted κ = 0.653), D2 simple agreement 80.0% (Cohen’s κ = 0.649; weighted κ = 0.720), D3 simple agreement 93.3% (Cohen’s κ = 0.857; weighted κ = 0.929), D4 simple agreement 66.7% (Cohen’s κ = 0.433; weighted κ = 0.564), and D5 100% agreement (Cohen’s κ = 1.000). Because several trials had “some concerns” or high risk in D1/D2 that could plausibly bias effect estimates, we downgraded once for risk of bias. |
| **Inconsistency** | **Not downgraded** | Although the initial multilevel meta-analysis indicated very high between-study heterogeneity (I² ≈ 93.3%) and the GOSH analysis (19,998 subsets) produced a wide distribution of effects (SMD range ≈ −1.47 to 0.73) with variable heterogeneity metrics (Tau² range 0.00–5.54 and very broad I² range), we conducted pre-specified and post-hoc robustness checks that materially addressed heterogeneity concerns. Specifically, we: (a) used GOSH plots to identify heterogeneous clusters and outlier subsets (≈6.40% flagged as outliers); (b) identified high-influence effect sizes by standardized residuals ( |
| **Indirectness** | **Not downgraded** | The population (college students), interventions (Chinese traditional mind-body practices such as Tai Chi, Baduanjin, Wuqinxi, Yijinjing, MTCC, etc.), comparators, and outcomes (validated depression scales: PHQ-9, SDS, CES-D, BDI, DASS-21 depression subscale, SCL-90-R depression subscale, etc.) directly addressed the review question; no serious indirectness was detected. |
| **Imprecision** | **Not downgraded** | The pooled estimate excluded the null and was clinically interpretable (SMD = −0.56, 95% CI −0.84 to −0.28). Trial sequential analysis indicated that the required information size (RSI = 42.17) had been exceeded (accumulated information ≈ 151.97, ≈ 3.6× RSI), and cumulative meta-analysis showed effect stability after 2023. These findings indicated sufficient precision and we therefore did not downgrade for imprecision. |
| **Publication bias** | **Not downgraded** | Formal small-study bias testing (Egger’s test) did not indicate statistically significant small-study effects (t = −1.325, P = 0.185). Multilevel trim-and-fill procedures suggested potential missing studies and produced an adjusted estimate of approximately SMD = −0.44 (absolute change 0.12; relative change 21.6%), but the adjusted effect remained statistically significant and directionally consistent with the primary result. Sensitivity analyses that excluded high-influence effects did not alter the substantive conclusion. Given that adjustments reduced but did not eliminate the effect and that multiple robustness checks supported the finding, we did not downgrade for publication bias. |
| **Overall quality of evidence** | **Moderate** | All included studies were randomized controlled trials (initial grading “high”). We downgraded one level for risk of bias (concerns concentrated in D1 and D2). Because heterogeneity was thoroughly investigated and influence diagnostics / sensitivity analyses (including exclusion of high-influence effects), robust variance estimation, and trial sequential analysis supported the stability and robustness of the pooled effect, we did not downgrade for inconsistency. We also did not downgrade for indirectness, imprecision, or publication bias. Consequently, the overall GRADE rating was **Moderate**. |

# Subgroup Analysis of Traditional Chinese Mind-Body Exercise on Depression Symptoms in University Students

| Dimensionality | sort | K | N | I^2^ | Effect model | SMD and 95%CI | GRADE | P | P-interaction |
| --- | --- | --- | --- | --- | --- | --- | --- | --- | --- |
| Intervention frequency/week |  |  |  |  |  |  |  |  | 0.298 |
|  | 1 | 3 | 351 | 93.0% | Random | -0.62(-1.59, 0.36) | Very Low | 0.215 |  |
|  | 2 | 2 | 122 | 0.0% | Random | -0.85(-1.23, -0.48) | Moderate | 0.001** |  |
|  | 3 | 5 | 575 | 79.4% | Random | -0.27(-0.78, 0.24) | Very Low | 0.305 |  |
|  | 5 | 4 | 255 | 0.0% | Random | -0.77(-1.03, -0.52) | Moderate | 0.001** |  |
| Intervention cycle/week |  |  |  |  |  |  |  |  | 0.001** |
|  | 1 | 1 | 80 | * | Random | -1.60(-2.11, -1.09) | Low | 0.001** |  |
|  | 8 | 3 | 140 | 0.0% | Random | -0.84(-1.18, -0.49) | Moderate | 0.001** |  |
|  | 10 | 2 | 156 | 82.6% | Random | -0.33(-1.25, 0.60) | Very Low | 0.482 |  |
|  | 12 | 4 | 287 | 87.8% | Random | -0.28(-1.04, 0.49) | Very Low | 0.476 |  |
|  | 16-18 | 4 | 640 | 0.0% | Random | -0.49(-0.64, -0.33) | Moderate | 0.001** |  |
| Intervention time/minute |  |  |  |  |  |  |  |  | 0.306 |
|  | 30-45 | 3 | 432 | 86.8% | Random | -0.96(-1.63, -0.30) | Low | 0.005* |  |
|  | 50-75 | 5 | 278 | 78.9% | Random | -0.27(-0.84, 0.30) | Very Low | 0.356 |  |
|  | 80-90 | 6 | 593 | 73.3% | Random | -0.56(-0.88, -0.23) | Moderate | 0.001** |  |
| Subject type |  |  |  |  |  |  |  |  | 0.860 |
|  | FUS | 3 | 218 | 82.7% | Random | -0.42(-1.10, 0.25) | Very Low | 0.219 |  |
|  | GUS | 3 | 391 | 85.7% | Random | -0.40(-0.93, 0.16) | Very Low | 0.164 |  |
|  | SDU | 2 | 130 | 0.0% | Random | -0.62(-0.97, -0.26) | Moderate | 0.001** |  |
|  | SSC | 6 | 564 | 81.7% | Random | -0.68(-1.23, -0.12) | Low | 0.018* |  |
| Intervention mode2 |  |  |  |  |  |  |  |  | 0.101 |
|  | Control-NoInt | 9 | 830 | 73.4% | Random | -0.72(-1.07, -0.38) | Moderate | 0.001** |  |
|  | Control-Int | 5 | 473 | 73.5% | Random | -0.29(-0.67, 0.10) | Very Low | 0.142 |  |
| Intervention mode1 |  |  |  |  |  |  |  |  | 0.023* |
|  | BDJ | 5 | 420 | 19.5% | Random | -0.64(-0.88, -0.40) | Moderate | 0.001** |  |
|  | QG | 1 | 29 | * | Random | 0.62(-0.13, 1.37) | Very Low | 0.103 |  |
|  | Tai Chi | 4 | 473 | 88.5% | Random | -0.64(-1.41, 0.14) | Very Low | 0.106 |  |
|  | Wuqinxi | 1 | 80 | * | Random | -0.57(-1.01, -0.12) | Low | 0.013* |  |
|  | Yijinjing | 1 | 120 | * | Random | -0.90(-1.28, -0.53) | Low | 0.001** |  |
|  | MTCC | 2 | 181 | 88.7% | Random | -0.37(-1.31, 0.58) | Very Low | 0.446 |  |
| Scale type |  |  |  |  |  |  |  |  | 0.009 |
|  | SDS | 4 | 430 | 0.0% | Random | -0.61(-0.84, -0.38) | Moderate | 0.001** |  |
|  | SCL-90-R | 6 | 511 | 82.4% | Random | -0.71(-1.18, -0.24) | Low | 0.003* |  |
|  | PHQ-9 | 2 | 214 | 58.1% | Random | -0.58(-1.05, -0.12) | Moderate | 0.014* |  |
|  | Other scales | 2 | 148 | 34.7% | Random | 0.25(-0.22, 0.73) | Very Low | 0.293 |  |
| ROB2_Domain |  |  |  |  |  |  |  |  | 0.705 |
|  | High | 1 | 80 | * | Random | -1.60(-2.11, -1.09) | Low | 0.001** |  |
|  | Some concerns | 11 | 1026 | 62.3% | Random | -0.50(-0.74, -0.26) | Moderate | 0.001** |  |
|  | Low | 2 | 197 | 91.3% | Random | -0.41(-1.41, 0.60) | Very Low | 0.429 |  |

**Note:** SMD, Standardized Mean Difference; statistical significance is defined as a 95% Confidence Interval (95% CI) that does not include zero. GRADE indicates the certainty of evidence (High, Moderate, Low, Very Low). P-interaction is the p-value for tests of subgroup differences. Subject subgroup definitions: FUS, Female University Students; GUS, General University Students; SDU, Sports-Disadvantaged University Students; SSC, University Students with Specific Constitutions (e.g., Qi-Stagnation constitution). Intervention modes: Control-NoInt, a control group receiving no active intervention; Control-Int, a control group receiving another active intervention (e.g., conventional exercise, health education). Specific traditional mind-body exercises: BDJ, Baduanjin; QG, Qigong; Tai Chi; Wuqinxi, Yijinjing; MTCC, Modified Tai Chi Chuan. Depression assessment scales: SDS, Self-Rating Depression Scale; SCL-90-R, Depression Symptom Checklist-90-Revised; PHQ-9, Patient Health Questionnaire-9-Depression subscale. ROB2, Risk of Bias 2 tool.

# Posterior Optimal Dose Distribution Plot

# Statistical model specification, diagnostics and reproducibility

Three-level synthesis. Effect sizes were nested within study, sampling variances were treated as known and within-study and between-study variance components were estimated by restricted maximum likelihood. Cluster-robust variance estimation used study as the clustering unit and a small-sample degrees-of-freedom correction. Leave-one-out and sequential dual-threshold influence diagnostics were used to evaluate sensitivity without relying on statistical significance alone.

Nonlinear and Bayesian analyses. Restricted cubic splines were evaluated only within the observed exposure range. Bayesian models used weakly informative priors and Markov chain Monte Carlo estimation. Convergence was assessed through trace-plot mixing, R-hat values and effective sample sizes; prior and knot-placement sensitivity analyses were used to assess dependence on modelling choices. Posterior medians, 95% credible intervals, posterior predictive intervals, density overlays and empirical cumulative-distribution comparisons are reported. The estimated 33.7-h minimum is a fitted, hypothesis-generating feature rather than an observed threshold or recommended dose.

Reproducibility and cross-referencing. The main Methods section describes the analytical sequence; this Supplementary Information provides the search strategies, agreement analyses, GOSH and influence diagnostics, trim-and-fill analysis, trial sequential analysis, GRADE rationale, subgroup results and Bayesian posterior checks. The extracted dataset, model specifications and R code are available from the corresponding author upon reasonable request; a permanent public repository has not yet been established.

Global Density Overlay (Observed yi vs. multiple yrep densities)

# Interval Plot: Showing posterior predictive intervals for each observation and plotting observed values

# ECDF Comparison
